# Genome-Wide Association Mapping of Chlorophyll Dynamics and Seed Weight Under Contrasting Nitrogen Conditions in Soybean

**DOI:** 10.3390/genes17080924

**Published:** 2026-08-06

**Authors:** Rudy Sleigh, Stella K. Kantartzi

**Affiliations:** Plant Genetic Improvement Lab, School of Agricultural Sciences, Southern Illinois University, Carbondale, IL 62901, USA; smith.sleigh@siu.edu

**Keywords:** soybean, genome-wide association study, SPAD, seed weight, BLUP, candidate genes, nitrogen-use efficiency

## Abstract

**Background**: Nitrogen (N) availability is an important factor affecting soybean growth, photosynthetic capacity, and seed development. Identification of genomic regions associated with N-responsive traits may enable the development of cultivars with enhanced performance under reduced fertilizer inputs. **Methods**: A soybean diversity panel consisting of 193 accessions was evaluated under N-deficient (N−) and N-sufficient (N+) conditions during the 2024 and 2025 growing seasons. Best linear unbiased predictors were estimated for SPAD area under the curve (SPAD AUC) and 1000-seed weight (SW) under N− and N+ conditions and used for genome-wide association analysis with 37,429 single-nucleotide polymorphism (SNP) markers. **Results**: Six loci associated with SPAD AUC under N−, four with SW under N−, and 10 with SW under N+ exceeded the Bonferroni-corrected significance threshold, whereas no significant loci were detected for SPAD AUC under N+. Candidate genes near significant loci encoded proteins including receptor-like kinases, WRKY transcription factors, peroxidases, and protein kinases, which are involved in signal transduction, transcriptional regulation, oxidative stress responses, carbohydrate metabolism, and developmental processes. Allelic effect analyses revealed significant phenotypic differences between favorable and unfavorable alleles at representative loci, supporting the biological relevance of the identified associations. **Conclusions**: These findings provide insight into the genetic architecture of chlorophyll accumulation and seed weight under contrasting N environments and identify candidate loci that may facilitate the improvement of nitrogen-use efficiency and agronomic performance in soybean.

## 1. Introduction

Soybean (*Glycine max* L. Merr.) is one of the world’s most important legume crops, serving as a major source of plant protein and oil for food, feed, and industrial applications [1]. As a legume, soybean can obtain a substantial portion of its nitrogen (N) requirements through biological nitrogen fixation (BNF), a symbiotic relationship with rhizobial bacteria in root nodules [2]. Nevertheless, N availability is a major factor influencing soybean growth, photosynthetic activity, and seed development. Environmental conditions that limit N acquisition can also reduce chlorophyll content, accelerate senescence, and negatively affect seed production [3,4].

Increased N-use efficiency has become an important objective in soybean improvement programs due to rising fertilizer costs and concerns about the environmental impacts of excessive N inputs [5]. Although soybeans can fix atmospheric N, considerable genetic variation exists among accessions in their ability to maintain productivity under contrasting N environments [6]. Identifying the genetic factors controlling these responses may facilitate the development of cultivars better adapted to low-input production systems while maintaining agronomic performance [7].

Genome-wide association studies (GWAS) have become a powerful technique for dissecting the genetic architecture of complex traits in soybean [8]. GWAS enables the identification of genomic regions and candidate genes associated with phenotypic variation at relatively high mapping resolution by exploiting recombination events across diverse germplasm panels [9]. Previous GWAS investigations have successfully identified loci controlling yield components, seed composition, abiotic stress responses, nodulation characteristics, and N-related traits; however, more studies have focused on traits evaluated under standard production systems [10,11,12,13,14,15]. Consequently, the genetic basis of chlorophyll dynamics and seed weight responses under contrasting N availability remains poorly understood in soybean.

Chlorophyll status is positively associated with plant N content and photosynthetic capacity, and thus, SPAD-based measurements are valuable indicators of N response [16]. Repeated SPAD measurements collected throughout the growing season can be integrated to obtain the area under the curve (AUC), providing a comprehensive assessment of chlorophyll dynamics over time [17]. Similarly, 1000-seed weight is an important yield component with relatively high genetic control that reflects seed filling and assimilate allocation under contrasting N availability. Its relatively stable genetic expression makes it well suited for identifying genomic regions associated with agronomic performance under different N conditions [18]. Nonetheless, dynamic N-responsive traits, such as SPAD AUC and seed weight responses to contrasting N supply, remain poorly characterized in soybean. Therefore, the objective of this study was to identify genomic regions and candidate genes associated with SPAD AUC and 1000-seed weight under N-deficient and N-sufficient conditions using a diverse soybean panel. Genome-wide association analyses were performed using BLUP-derived phenotypes, and significant marker–trait associations were further investigated through candidate gene annotation and allelic effect analyses. The results provide new insights into the genetic architecture of soybean responses to N availability and identify loci that may facilitate marker-assisted selection and the improvement of N-use efficiency.

## 2. Materials and Methods

### 2.1. Plant Materials and Field Evaluation

A soybean (*Glycine max* L. Merr.) diversity panel consisting of 193 accessions representing maturity groups (MG) III–VII was evaluated in this study (Table S1). The panel originated from 12 countries and included 135 accessions from China, 17 from Japan, 15 from South Korea, 14 from the United States, 3 from Taiwan, 2 from India, 2 from Uganda, and one accession each from Georgia, Morocco, Nepal, South Africa, and Vietnam. Accessions were selected from the USDA Soybean Germplasm Collection based on their origin and overall agronomic performance. Field experiments were conducted during the 2024 and 2025 growing seasons at the Agricultural Research Center, Southern Illinois University, Carbondale, IL, USA (37.7° N, 89.2° W). The experimental site is representative of soybean production systems in southern Illinois and consists of silt loam soils developed from loess deposits. Soybeans were planted following corn under a no-till production system. The experiment was arranged as a randomized complete block design with two N treatments: a non-fertilized treatment (N−) and a fertilized treatment (N+). Within each N treatment, soybean accessions were randomly assigned to plots within each of two replications to minimize the effects of field variability. Experimental units consisted of two-row plots measuring 4.57 m in length with 0.76 m row spacing. Seeds were planted at a density of 37 seeds m^−2^ to a depth of approximately 2.5 cm. In the N+ treatment, 70.61 kg N ha^−1^ was applied as urea immediately after planting, whereas no N fertilizer was applied in the N− treatment. This application rate was selected to provide a non-limiting N supply and establish contrasting N environments that would maximize phenotypic differences among accessions. Standard agronomic practices recommended for soybean production in southern Illinois were followed throughout both growing seasons, including weed management and routine monitoring for pests and diseases. No supplemental irrigation was applied. No commercial rhizobial inoculant was applied because the experimental field has a long history of soybean cultivation and was assumed to contain established native *Bradyrhizobium japonicum* populations capable of supporting nodulation.

### 2.2. Phenotypic Data Collection

Leaf chlorophyll content was measured using a SPAD meter (SPAD-502, Konica Minolta, Tokyo, Japan). Measurements were collected from the first trifoliate (V1) through the full seed (R6) developmental stages. SPAD readings were recorded twice weekly, resulting in 30 sampling time points (T1–T30) per growing season. Measurements were obtained between 0800 and 1000 h under clear weather conditions to minimize environmental variation. At each sampling time point, SPAD measurements were collected from three fully expanded upper-canopy leaves on each of three randomly selected plants within each plot. SPAD values were averaged to generate a mean for each accession, treatment, and sampling time point. Chlorophyll dynamics were quantified by calculating SPAD AUC, expressed as SPAD units × days, using the trapezoidal integration method:

$$SPAD\;AUC=\sum_{T=1}^{n-1}\left(\frac{{SPAD}_{i}+\;{SPAD}_{i+1}}{2}\right)(T_{i+1}-T_{i})$$

where SPADi and SPADi+1 represent SPAD measurements at consecutive sampling times, and Ti and Ti+1 represent the corresponding sampling dates. At physiological maturity (R8), plots were harvested, and seed samples were cleaned and dried prior to determining 1000-seed weight (g) using a precision balance. SPAD AUC and 1000-seed weight were subsequently used for estimation of best linear unbiased predictor (BLUP) values and GWAS.

### 2.3. Estimation of BLUP Values

BLUP values were estimated separately for each trait and N treatment using mixed-model analysis in JMP 19.0 (SAS Institute Inc., Cary, NC, USA). Analyses were conducted independently for SPAD AUC and 1000-seed weight under N− and N+ conditions. For each trait-treatment combination, year was fitted as a fixed effect, and accession was fitted as a random effect according to the following model:

$$Y_{ij}=\mu +Y_{i}+G_{j}+\varepsilon_{ij}$$

where Yij is the observed phenotypic value, μ is the overall mean, Yi is the fixed effect of year, Gj is the random effect of accession, and εij is the residual error. Variance components were estimated using restricted maximum likelihood (REML). Treatment-specific accession BLUP values were extracted for SPAD AUC under N− and N+ conditions and for 1000-seed weight under N− and N+ conditions. The resulting BLUP values were used as phenotypic inputs for all subsequent statistical analyses, including trait correlation analysis and GWAS. Broad-sense heritability (H^2^) was estimated from variance components obtained from a combined analysis across the four experimental environments (two years × two nitrogen treatments) according to:

$$H^{2}\;=\;\frac{\sigma^{2}g}{\sigma^{2}g\;+\;\frac{\sigma^{2}e}{r}}$$

where σ^2^g is the genotypic variance, σ^2^e is the residual variance, and r is the number of environments included in the analysis.

Phenotypic data were analyzed using JMP 19.0 (SAS Institute Inc., Cary, NC, USA). Descriptive statistics, including means, standard deviations, ranges, and coefficients of variation (CV), were calculated for all traits. Pearson correlation coefficients were calculated among the four BLUP-derived phenotypes, including SPAD AUC under N− conditions (SPAD AUC N−), SPAD AUC under N+ conditions (SPAD AUC N+), 1000-seed weight under N− conditions (SW N−), and 1000-seed weight under N+ conditions (SW N+).

### 2.4. Genotypic Data and Marker Filtering

Genome-wide single-nucleotide polymorphism (SNP) data for the soybean accessions were obtained from SoyBase (https://soybase.org; accessed on 1 June 2026), where genotypic information generated using the SoySNP50K iSelect BeadChip (Illumina Inc., San Diego, CA, USA) is publicly available [19]. SNP quality control filtering was performed using PLINK 1.9 [20]. Markers with a minor allele frequency (MAF) less than 0.05, missing genotype data exceeding 20%, or heterozygosity greater than 20% were removed. After filtering, 37,429 high-quality SNP markers across 193 soybean accessions were retained for subsequent population structure and genome-wide association analyses.

### 2.5. Population Structure Analysis

Population structure within the soybean diversity panel was evaluated using principal component analysis (PCA) and model-based ancestry estimation. PCA was performed using PLINK 1.9 to assess genetic relationships among accessions and identify the major axes of genetic variation. Population ancestry was further inferred using ADMIXTURE 1.3.0 [21]. Cross-validation errors were calculated for K values ranging from 1 to 10 to estimate the most likely number of genetic subpopulations within the panel. The optimal number of ancestral populations was determined as the K value associated with the lowest cross-validation error. The ADMIXTURE cross-validation error plot, principal component analysis (PCA) scatter plot, and ancestry coefficient bar plot were generated using R (R Core Team, Vienna, Austria).

### 2.6. GWAS

Association analyses were performed using GAPIT 3 with the Fixed and Random Model Circulating Probability Unification (FarmCPU) model [22]. The first three principal components were included as covariates to account for population structure, and FarmCPU was used to control for confounding between marker effects and population-relatedness. Marker-trait associations surpassing the Bonferroni-corrected significance cutoff (*p* < 1.34 × 10^−6^; −log10(*p*) > 5.87) were considered significant. Because FarmCPU is a multi-locus GWAS method, marker effect sizes and phenotypic variance explained are not directly estimated in a manner comparable to single-marker regression models. Therefore, allelic effects of representative significant SNPs were evaluated to illustrate their phenotypic impact.

### 2.7. Candidate Gene Identification

Candidate genes associated with significant marker-trait associations were identified using the soybean reference genome annotation available through SoyBase (https://soybase.org; accessed 11 June 2026). For each significant SNP identified by GWAS, genes located within a ±100 kb genomic window surrounding the peak marker were examined. This window was selected to capture genes in close physical proximity to significant SNPs while limiting the inclusion of unrelated genes. Because linkage disequilibrium varies among soybean populations and genomic regions, the identified genes should be regarded as putative candidates requiring further validation. Functional annotations predicted protein domains, and Gene Ontology (GO) terms from SoyBase were used to assess the potential biological relevance of candidate genes.

### 2.8. Allelic Effect Analysis

Allelic effects of selected significant SNP markers were evaluated by grouping accessions according to SNP genotype and comparing the corresponding BLUP values. SNP markers selected for allelic effect analysis were chosen based on a combination of statistical significance, biological relevance of nearby candidate genes, balanced allele frequencies, and clear phenotypic separation between allelic classes. Genotype frequencies were calculated for each SNP using non-missing genotype calls. Phenotypic distributions among allelic classes were visualized using violin plots with overlaid boxplots and individual observations. Statistical differences between allelic classes were evaluated using two-sample *t*-tests in JMP 19.0.

## 3. Results

### 3.1. Phenotypic Variation, Heritability, and BLUP Distributions

**Figure 1:**
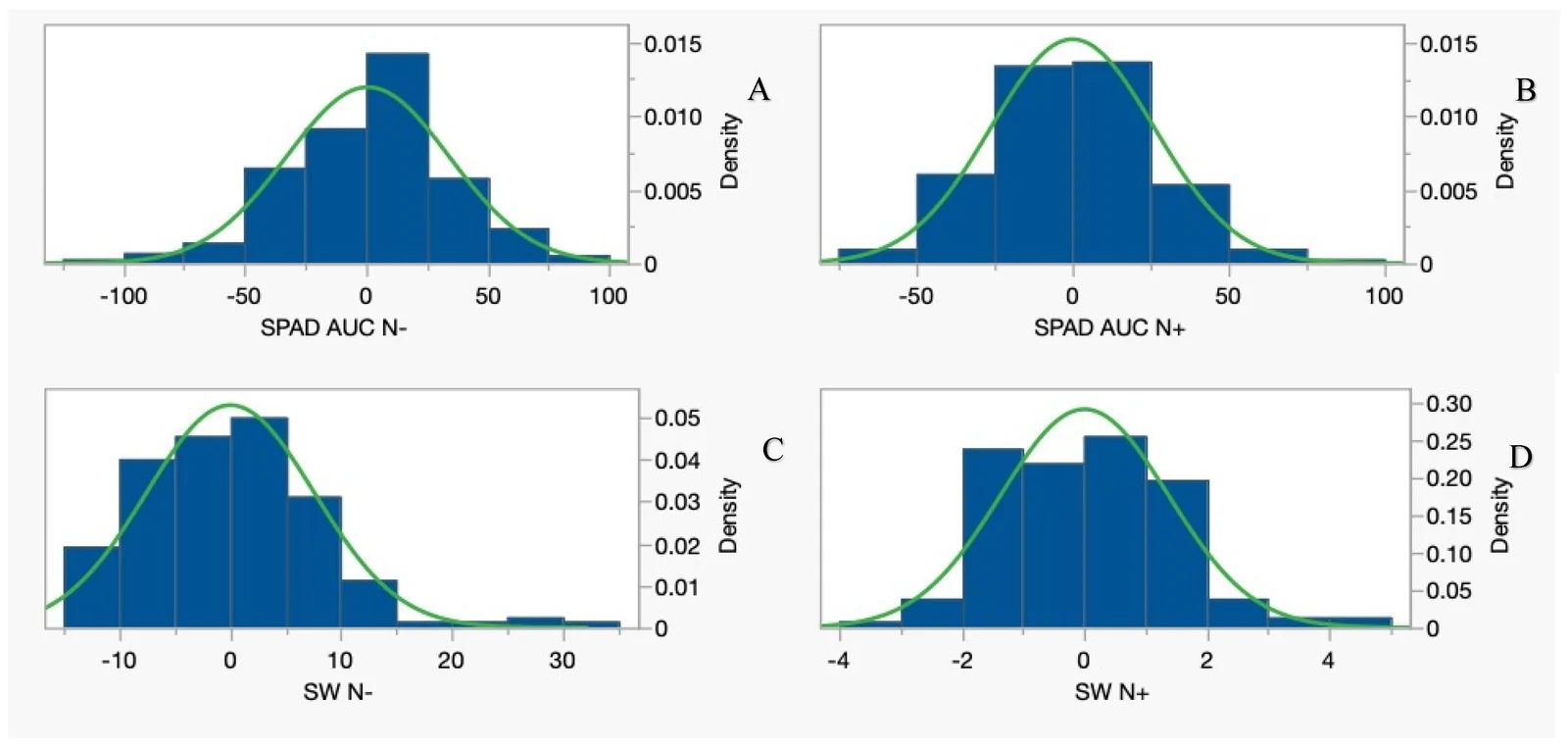
Distribution of best linear unbiased predictor (BLUP) values for (**A**) SPAD area under the curve under nitrogen-deficient conditions (SPAD AUC N−). (**B**) SPAD AUC under nitrogen-sufficient conditions (SPAD AUC N+). (**C**) 1000-seed weight under N− (SW N−) (**D**) SW N+.

Substantial phenotypic variation was observed among the 193 soybean accessions for both SPAD AUC and SW under N− and N+ conditions (Table 1). Mean SPAD AUC was 728.78 under N− and 782.88 under N+, whereas mean SW was 116.51 g under N− and 118.94 g under N+. SPAD AUC ranged from 144.80 to 994.25 under N− and from 564.75 to 1065.15 under N+, whereas SW ranged from 35.9 to 278.5 g under N− and from 42.6 to 287.2 g under N+. CV was greater for SW (28.99–29.89%) than for SPAD AUC (12.51–17.81%), indicating greater relative phenotypic diversity for SW. Broad-sense heritability estimates were 0.69 for SPAD AUC and 0.59 for SW, displaying moderate to high genetic control of both traits. To account for environmental variation across years, BLUP values were estimated separately for each trait and N environment. The resulting BLUP distributions exhibited continuous variation for all four trait-environment combinations (Figure 1), supporting the suitability of these traits for GWAS.

### 3.2. Relationship Between SPAD AUC and SW Under N− and N+

**Figure 2:**
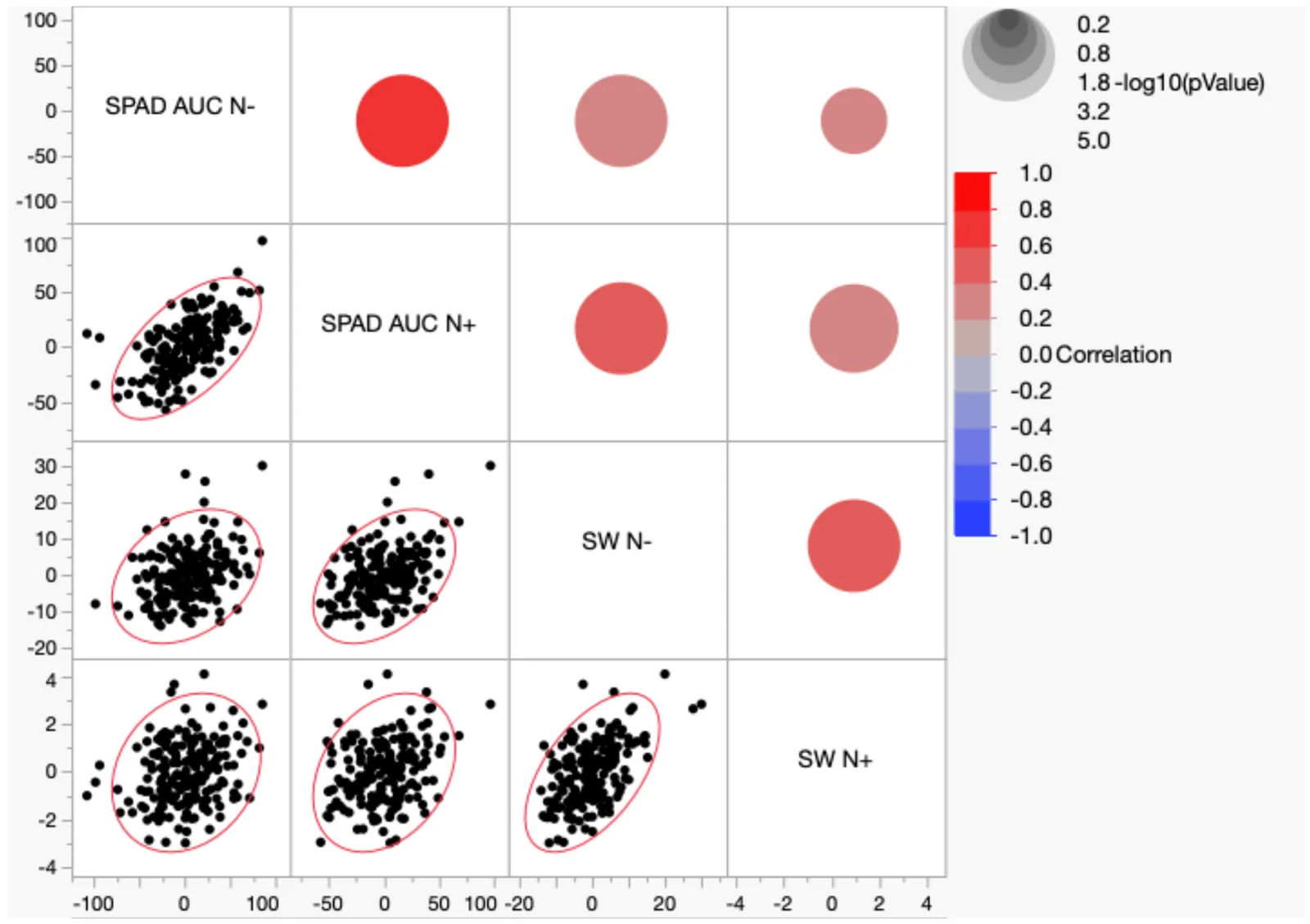
Pearson correlation analysis among BLUP-derived SPAD area under the curve (SPAD AUC) and 1000-seed weight (SW) traits under nitrogen-deficient (N−) and nitrogen-sufficient (N+) conditions. Circle size and shading are proportional to the magnitude of the correlation coefficient, with larger circles indicating stronger correlations. Corresponding correlation coefficients and *p*-values are presented in the upper and lower panels, respectively.

Pearson correlation analysis revealed significant positive relationships among all four BLUP traits (Figure 2). Because the analysis was performed using BLUPs, the resulting correlation coefficients describe relationships among predicted genetic values after accounting for environmental variation rather than among raw phenotypic observations. The strongest correlation was observed between SPAD AUC N− and SPAD AUC N+ (*r* = 0.596, *p* < 0.0001). Similarly, SW N− and SW N+ showed a strong positive correlation (*r* = 0.569, *p* < 0.0001). Additionally, SW N− was moderately correlated with SPAD AUC N+ (*r* = 0.442, *p* < 0.0001) and SPAD AUC N− (*r* = 0.328, *p* < 0.0001). Likewise, SW N+ was positively associated with SPAD AUC N+ (r = 0.299, *p* < 0.0001) and SPAD AUC N− (r = 0.228, *p* = 0.0017).

### 3.3. Population Structure of the Soybean Diversity Panel

**Figure 3:**
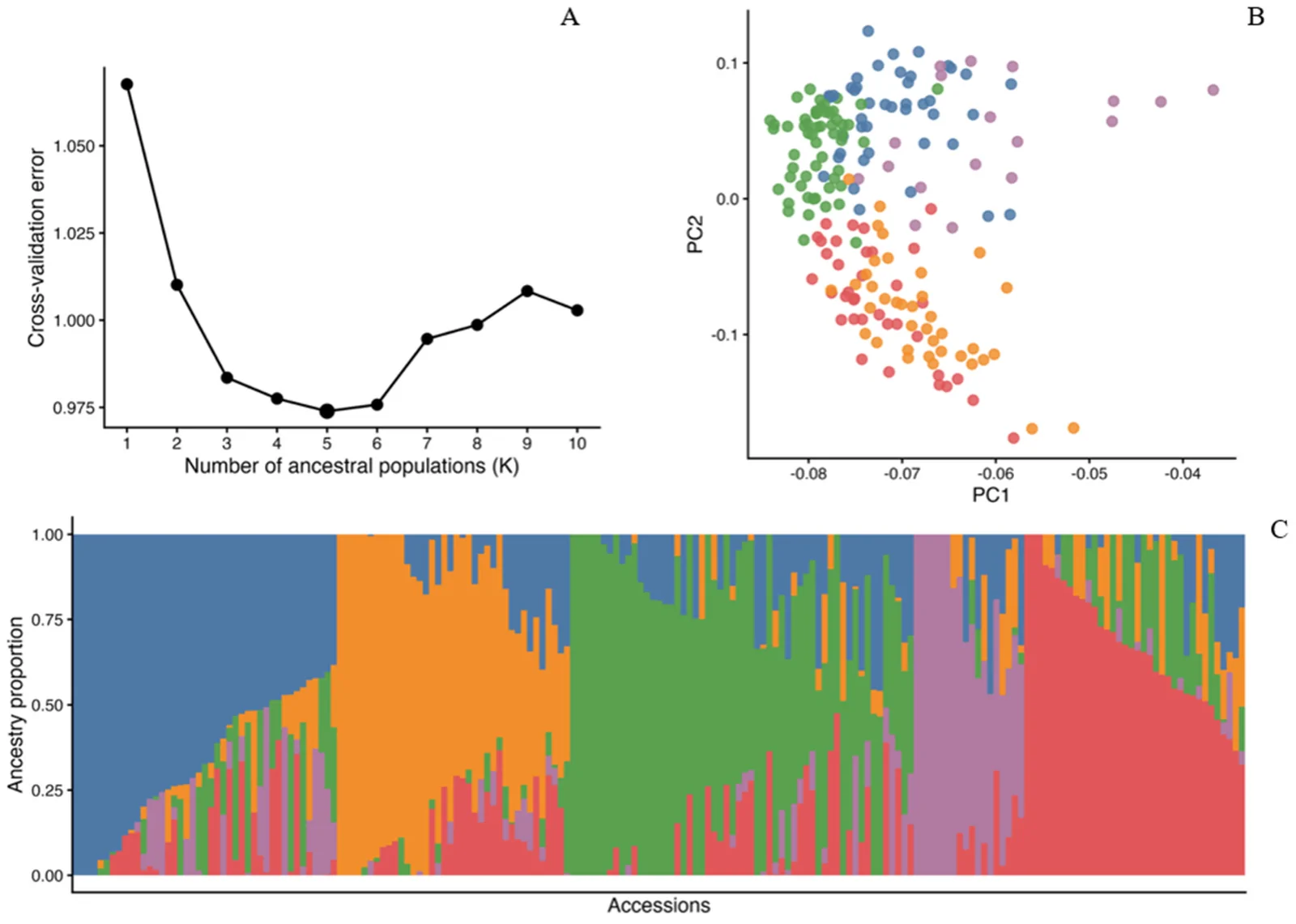
Population structure of the soybean diversity panel based on 37,429 SNP markers. (**A**) ADMIXTURE cross-validation error for K = 1–10 ancestral populations. The lowest cross-validation error was observed at K = 5, indicating the most likely number of subpopulations. (**B**) Principal component analysis (PCA) of 193 soybean accessions based on the first two principal components (PC1 and PC2). Colors represent the five subpopulations inferred by ADMIXTURE. (**C**) ADMIXTURE ancestry coefficients for K = 5. Each vertical bar represents an accession, and colors indicate the proportion of ancestry derived from each inferred subpopulation.

ADMIXTURE analysis identified K = 5 as the most probable number of ancestral populations, based on the lowest cross-validation error (Figure 3A). The gradual decrease in cross-validation error from K = 1 to K = 5, followed by stabilization at higher K values, supported the presence of five genetic subpopulations within the panel. PCA further revealed genetic stratification among the 193 soybean accessions (Figure 3B). Although clustering identified five subpopulations, overlap among groups was evident, indicating substantial admixture within the panel. The first two principal components captured the major axes of genetic variation and provided a visual representation of genetic relationships among accessions. ADMIXTURE ancestry coefficients confirmed the presence of five subpopulations and revealed varying levels of admixture among accessions (Figure 3C).

### 3.4. Genome-Wide Association Analysis

**Figure 4:**
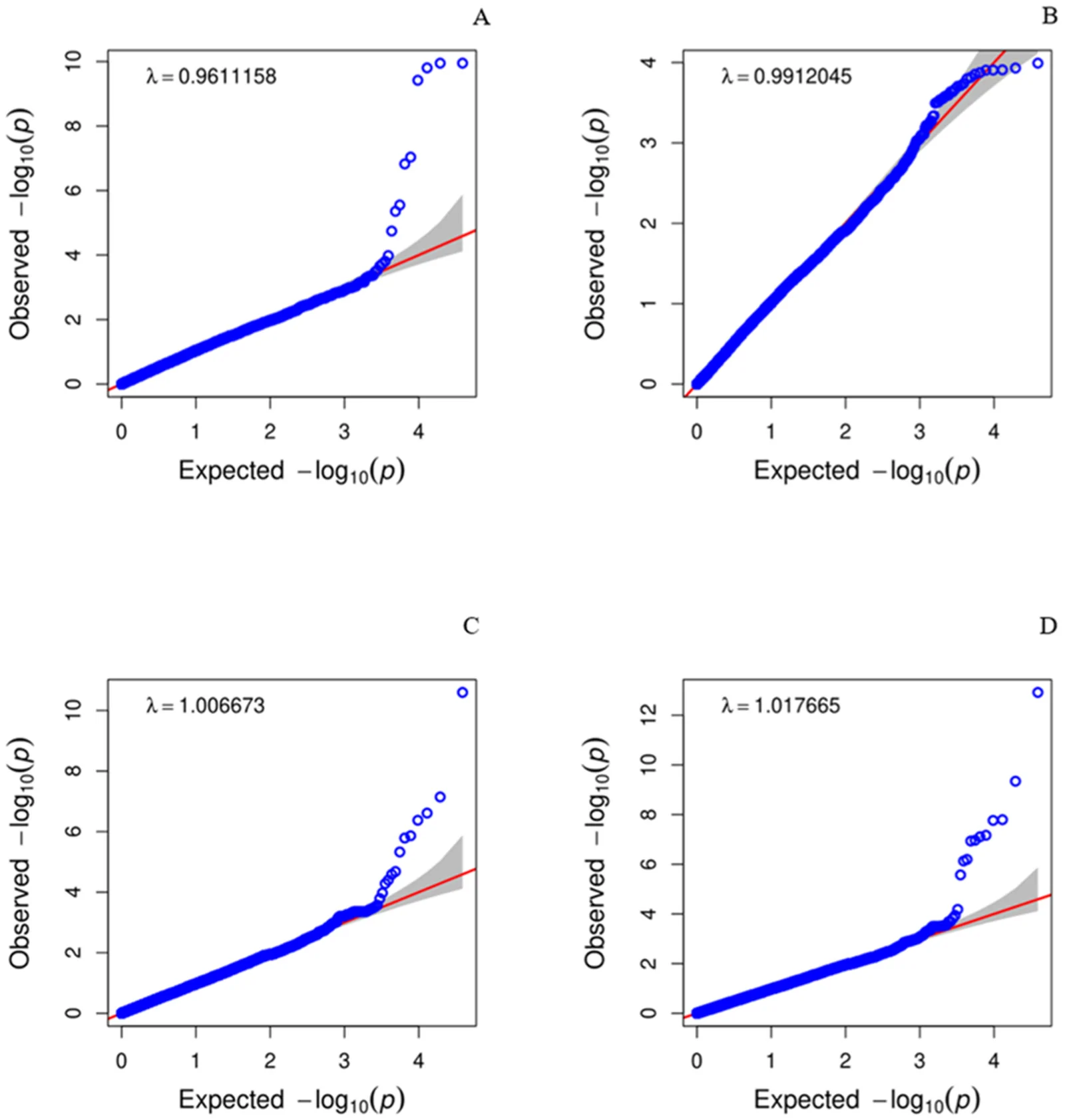
Quantile–quantile plots of genome-wide association analyses for (**A**) SPAD area under the curve under nitrogen-deficient conditions (SPAD AUC N−), (**B**) SPAD AUC under nitrogen-sufficient conditions (SPAD AUC N+), (**C**) 1000-seed weight under N− (SW N−), and (**D**) SW N+.

**Figure 5:**
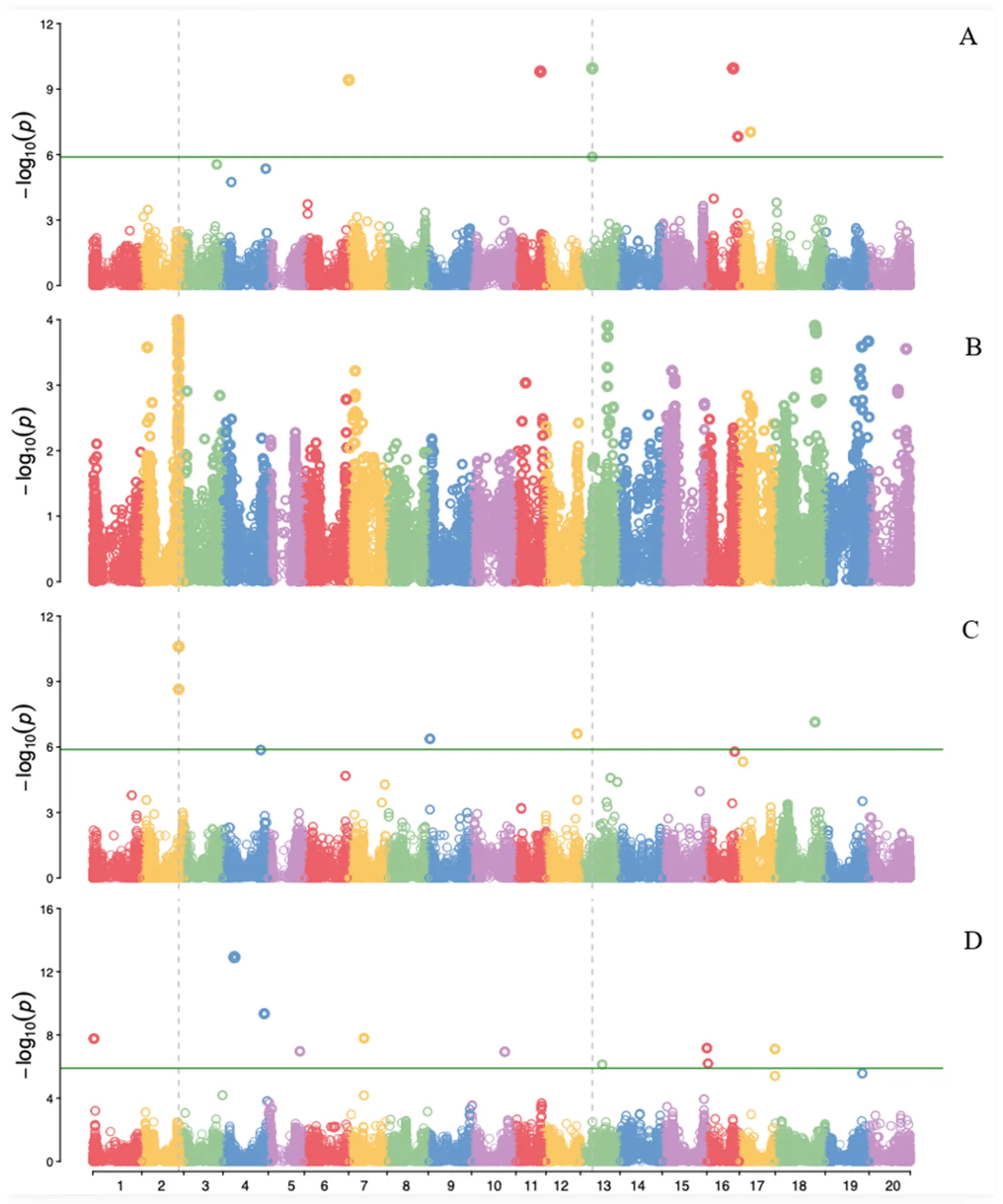
Manhattan plots of genome-wide association analyses for (**A**) SPAD area under the curve under nitrogen-deficient conditions (SPAD AUC N−), (**B**) SPAD AUC under nitrogen-sufficient conditions (SPAD AUC N+), (**C**) 1000-seed weight under N− (SW N−), and (**D**) SW N+. The horizontal line indicates the significance threshold, and SNPs above the threshold were considered significantly associated with the corresponding trait.

Quantile–quantile plots demonstrated effective control of population structure and relatedness, with genomic inflation factors (λ) ranging from 0.96 to 1.02 across traits (Figure 4). The close correspondence between observed and expected *p*-value distributions indicated minimal inflation and a low frequency of false-positive associations, while deviations in the upper tail of the distributions reflected true marker-trait associations. Manhattan plots revealed distinct genomic regions associated with SPAD AUC and SW under N− and N+ (Figure 5). For SPAD AUC N−, six significant loci were identified on chromosomes (Chr.) 7, 11, 13, 16, and 17. The strongest associations corresponded to ss715624313 on Chr. 16 (−log10(*p*) = 9.95), ss715617226 on Chr. 13 (−log10(*p*) = 9.95), ss715609999 on Chr. 11 (−log10(*p*) = 9.80), and ss715596121 on Chr. 7 (−log10(*p*) = 9.42). Additional significant loci were identified at ss715625979 on Chr.17 and ss715624813 on Chr. 16. For SW N−, four significant loci were identified on Chr. 2, 9, 12, and 18. The most significant association was observed for ss715582932 on Chr. 2 (−log10(*p*) = 10.60), followed by loci on Chr. 18, 12, and 9. For SW under N+ conditions, 10 significant SNP markers were identified on Chr. 1, 4, 5, 7, 10, 13, 16, and 17. The strongest association corresponded to ss715587094 on Chr. 4, which exhibited a −log10(*p*) value of 12.92. Additional highly significant loci included ss715588530 on Chr. 4 (−log10(*p*) = 9.34), ss715596677 on Chr. 7 (−log10(*p*) = 7.79), and ss715578470 on Chr. 1 (−log10(*p*) = 7.77) (Table 2).

### 3.5. Candidate Genes Associated with Significant Marker-Trait Associations

Functional annotations revealed several candidate genes with known or predicted roles in signal transduction, transcriptional regulation, stress responses, chlorophyll maintenance, carbon metabolism, and seed development (Table 3). For SPAD AUC N−, candidate genes included *Glyma.07G147900*, encoding a leucine-rich repeat (LRR) receptor-like kinase, a class of proteins involved in environmental signal perception and phosphorylation-mediated signaling; *Glyma.17G153400*, a protein kinase superfamily member potentially involved in growth regulation and nutrient-response pathways; *Glyma.16G200300*, a peroxidase superfamily protein with potential functions in reactive oxygen species detoxification and oxidative-stress tolerance, processes that may contribute to chlorophyll maintenance under N limitation; *Glyma.11G202600*, a 2-oxoglutarate and Fe(II)-dependent oxygenase, which may participate in N and carbon metabolism, and *Glyma.13G044600*, an alpha/beta hydrolase, potentially involved in stress-responsive metabolic pathways. For SW N−, candidate genes included *Glyma.02G240100*, an acyl-CoA synthetase 5, which functions in fatty acid metabolism and carbon allocation during seed development; *Glyma.18G191100*, a MYB/SANT-like DNA-binding protein involved in transcriptional regulation; *Glyma.12G202700*, an EPIDERMAL PATTERNING FACTOR 1-like protein with potential roles in developmental signaling and resource-use efficiency; and *Glyma.09G023600*, a homeodomain/START-domain DNA-binding protein implicated in developmental regulation and stress responses. For SW N+, candidate genes were associated with stress, defense, and nutrient-response pathways (*Glyma.04G115500*); phosphoinositide signaling (*Glyma.04G206100*); starch metabolism and carbohydrate partitioning (*Glyma.07G148000*); receptor kinase signaling (*Glyma.01G013500*); ethylene-responsive transcriptional regulation (*Glyma.05G179900*); phospholipid-mediated stress signaling (*Glyma.10G150200*); and protein phosphorylation cascades (*Glyma.13G139800*).

### 3.6. Allelic Effects of Major SNP Markers Associated with SPAD AUC and SW

**Figure 6:**
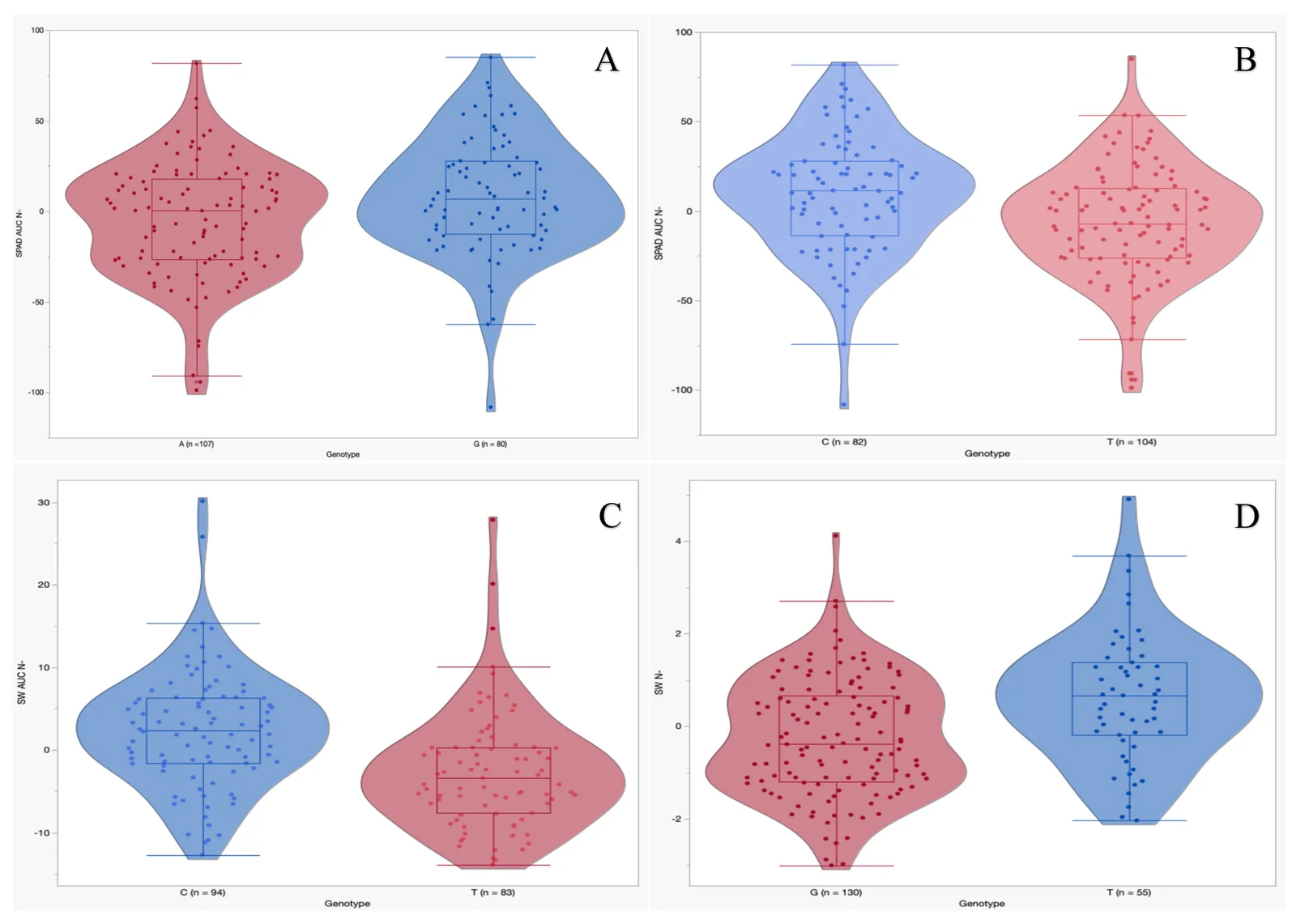
Allelic effects of (**A**) ss715596121 associated with SPAD area under the curve (SPAD AUC) under nitrogen-deficient conditions (N−); (**B**) ss715624813 associated with SPAD AUC under N−; (**C**) ss715612654 associated with 1000-seed weight (SW) under N−; and (**D**) ss715614171 associated with 1000-seed weight under nitrogen-sufficient conditions (N+). Violin plots show the distribution of BLUP values for each allelic class, and boxplots represent median and interquartile ranges.

To further evaluate the phenotypic effects of significant loci, allelic effects were examined for four representative SNP markers associated with SPAD AUC N− (ss715596121 and ss715624813), SW N− (ss715612654), and SW N+ (ss715614171) (Figure 6). These SNP markers were selected based on statistical significance and the biological relevance of their associated candidate genes. Genotype frequencies ranged from 55 to 130 accessions per allelic class, indicating adequate representation of both alleles within the diversity panel. For SPAD AUC N−, significant differences were observed between allelic classes at both loci. At ss715596121, accessions carrying the G allele exhibited higher BLUP values (mean = 8.57) than those carrying the A allele (mean = −5.29), corresponding to an approximate difference of 13.9 SPAD AUC units (*p* = 0.0057) (Figure 6A). Similarly, at ss715624813, the C allele was associated with higher SPAD AUC values (mean = 9.49) than the T allele (mean = −6.18), indicating a favorable effect of the C allele under N− (*p* = 0.00076) (Figure 6B). For SW N−, ss715612654 exhibited a pronounced allelic effect since accessions carrying the C allele had greater SW BLUP values (mean = 2.36) than those carrying the T allele (mean = −2.59), representing a difference of approximately 4.95 units (*p* = 7.6 × 10^−7^) (Figure 6C). For SW N+, accessions carrying the T allele of ss715614171 displayed significantly higher (*p* = 0.00018) BLUP values (mean = 0.62) than those carrying the G allele (mean = −0.25) (Figure 6D).

## 4. Discussion

### 4.1. Genetic Variation and Heritability

The soybean diversity panel showed substantial phenotypic variation for both SPAD AUC and SW under N− and N+ conditions. The broad ranges found across all traits, together with moderate-to-high heritability estimates, revealed considerable genetic diversity. Broad-sense heritability estimates of 0.69 for SPAD AUC and 0.59 for SW suggest that a substantial proportion of the observed phenotypic variation was attributable to genetic effects rather than environmental effects [10,13]. These values are consistent with previous reports demonstrating moderate to high heritability for chlorophyll-related traits and seed weight in soybean, supporting the suitability of these traits for association mapping and marker-assisted breeding [13,23,24,25,26]. N availability had a pronounced effect on chlorophyll accumulation, as reflected by the lower mean SPAD AUC N−. N is a major structural component of chlorophyll molecules and photosynthetic enzymes; therefore, its limitation often leads to reduced chlorophyll content and accelerated leaf senescence [27]. The lower mean SPAD AUC under N− conditions demonstrates the sensitivity of this trait to N availability and supports its value as an indicator of plant N status and photosynthetic performance. In contrast, SW showed only modest differences in mean values between N− and N+ conditions despite substantial phenotypic variation among accessions within each treatment. This indicates that seed weight was influenced primarily by genetic differences among accessions rather than by N availability alone. Seed weight is therefore best interpreted as a genetically controlled yield-component trait evaluated under contrasting N environments rather than as a direct physiological indicator of N response. Nevertheless, evaluating this trait separately under N− and N+ conditions enabled the identification of genomic regions associated with agronomic performance under contrasting N availability. The maintenance of seed weight under stress conditions has been reported previously and is often associated with efficient remobilization of stored N reserves from vegetative tissues to developing seeds [28]. The observed genetic variation in seed therefore provides opportunities to identify loci associated with N-use efficiency and yield stability. The combination of extensive phenotypic diversity and moderate-to-high heritability observed in this study provided a strong basis for GWAS. These results show that both chlorophyll accumulation and seed weight are under substantial genetic control and can be effectively used to identify genomic regions that contribute to soybean adaptation to contrasting N environments.

### 4.2. Biological Interpretation of the Relationship Between SPAD AUC and SW Under N− and N+

Correlation analysis indicated significant positive relationships among all BLUP-derived traits, indicating that chlorophyll accumulation and seed weight are connected components in soybean under N− and N+ conditions. The strongest correlations were observed between SPAD AUC N− and N+ (0.596), as well as between SW N− and N+ (0.569), denoting a degree of stability in the genetic factors controlling these traits. Positive correlations were also observed between SPAD AUC and SW across N environments. Accessions with higher chlorophyll accumulation tended to produce heavier seeds, supporting the relationship between photosynthetic capacity and yield formation. Chlorophyll content is an indicator of leaf N status and photosynthetic activity, both of which influence carbon assimilation during vegetative growth and reproductive development. Consequently, genotypes capable of retaining higher chlorophyll levels may sustain greater photosynthetic productivity, thereby providing additional assimilates for seed filling [29]. The association between SPAD AUC and seed weight was especially clear under N− conditions, where genetic differences in N acquisition, assimilation, and remobilization are expected to become more pronounced. N deficiency accelerates leaf senescence and chlorophyll degradation, consequently reducing photosynthetic capacity and ultimately limiting the availability of assimilates for reproductive growth [30]. Genotypes that maintain chlorophyll accumulation under stress conditions may therefore display better N-use efficiency and greater capacity to sustain seed development despite reduced N availability. These results support the use of SPAD AUC as an indicator of N-responsive performance in soybean. The positive relationship between chlorophyll accumulation and seed weight suggests that loci affecting chlorophyll maintenance may indirectly contribute to yield stability under N-limited conditions. Consequently, identifying genomic regions associated with SPAD AUC may provide valuable insights into the genetic mechanisms underlying N-use efficiency and adaptation to reduced N inputs.

### 4.3. Genomic Regions Associated with SPAD AUC and SW Under N− and N+

GWAS identified multiple loci associated with chlorophyll accumulation and seed weight, demonstrating that these traits are under complex genetic control. Significant marker-trait associations were detected for SPAD AUC N−, SW N−, and SW N+, whereas no loci surpassed the significance threshold for SPAD AUC N+, which may indicate that chlorophyll accumulation is less genetically differentiated when N availability is adequate. Under N sufficiency, physiological requirements for chlorophyll synthesis are mostly met, potentially reducing phenotypic contrasts among accessions [31]. Although the mean 1000-seed weight differed only slightly between N− and N+ conditions, substantial variation was observed among soybean accessions within each treatment. This indicates that seed weight was influenced primarily by genetic differences among accessions rather than by N treatment alone. Consequently, 1000-seed weight should be interpreted as a genetically controlled yield-component trait evaluated under contrasting N environments rather than as a direct physiological indicator of N response. Nevertheless, conducting separate GWAS analyses under each N condition enabled the identification of genomic regions associated with seed weight in each environment, providing insight into the genetic architecture underlying agronomic performance under contrasting N availability. In contrast, N deficiency imposes a physiological constraint that can uncover underlying genetic differences in N acquisition, assimilation, remobilization, and stress adaptation [32]. Similar observations have been reported in other plant species, where stress environments often reveal loci that remain undetected under optimal growing conditions [33,34]. Several loci associated with SPAD AUC N− were identified on Chr. 7, 11, 13, 16, and 17, suggesting that chlorophyll maintenance under stress is governed by multiple genomic regions distributed throughout the soybean genome. Likewise, SW N− and SW N+ were associated with distinct loci, indicating that different genomic regions were detected under the two N environments. Nevertheless, evaluating germplasm under contrasting N conditions enabled the identification of loci that may contribute to adaptation under specific N environments. The identification of multiple significant loci supports the quantitative nature of both chlorophyll accumulation and seed weight. Because GWAS analyses were conducted separately within each N treatment, these findings should be interpreted as treatment-specific associations rather than formal evidence of differential genetic mechanisms or genotype × N interactions. Therefore, loci detected under N-deficient conditions are of particular interest, as they may directly contribute to improved performance when N availability is limited.

### 4.4. Biological Significance of Candidate Genes and Allelic Effects

The candidate genes identified in this study suggest that soybean responses to contrasting N environments are influenced by a diverse network of signaling, transcriptional regulation, oxidative stress response, and developmental pathways. Although functional validation will be required to confirm the roles of these genes, their annotated functions provide biologically plausible explanations for the observed marker-trait associations. Among the loci associated with SPAD AUC N−, several candidate genes were related to signal perception and stress adaptation. The high-priority candidate *Glyma.07G147900*, located near ss715596121, encodes an LRR receptor-like kinase that plays central roles in environmental sensing and in activating downstream signaling cascades in response to abiotic and biotic stimuli [35]. Similarly, *Glyma.17G153400* and *Glyma.13G139800* encode protein kinases involved in phosphorylation-mediated signal transduction, a fundamental mechanism regulating plant growth, nutrient responses, and stress adaptation [36]. The recurrent identification of kinase-related genes among significant loci suggests that signaling networks may contribute substantially to variation in N-responsive traits. Additionally, the peroxidase superfamily gene *Glyma.16G200300*, located near ss715624813, is particularly noteworthy because peroxidases are involved in the detoxification of reactive oxygen species and in protection against oxidative damage. N deficiency is often accompanied by oxidative stress and accelerated chlorophyll degradation, making antioxidant-related genes attractive candidates for maintaining photosynthetic activity under nutrient limitation [37]. Several candidate genes associated with seed weight are involved in developmental regulation and assimilate partitioning. *Glyma.04G115500* was located near the most significant locus detected for SW N+ encoding WRKY proteins that are widely recognized as regulators of stress responses, hormone signaling, and nutrient-responsive gene expression [38]. Allelic effect analyses further supported the biological relevance of the identified loci. Representative SNP markers were selected for allelic effect analysis because they combined strong statistical support with biologically relevant candidate genes and well-balanced genotype classes, allowing meaningful evaluation of phenotypic differences between alternative alleles. Significant phenotypic differences were observed between alternative alleles for all four representative SNP markers examined. Accessions carrying favorable alleles exhibited increased SPAD AUC or SW relative to alternative allelic classes, with statistical significance ranging from *p* = 0.0057 to *p* = 7.6 × 10^−7^. These results indicate that the detected loci contribute measurable phenotypic effects and may represent useful targets for marker-assisted selection. In particular, the favorable alleles at ss715596121 and ss715624813 were associated with improved chlorophyll accumulation under N− conditions, whereas favorable alleles at ss715612654 and ss715614171 were associated with increased seed weight. The consistency between GWAS signals, candidate gene annotations, and allelic effect analyses strengthens confidence in the biological relevance of these genomic regions. Collectively, the candidate gene and allelic effect analyses suggest that soybean adaptation to contrasting N environments is governed by interconnected pathways involving environmental sensing, signal transduction, and oxidative stress protection. These findings provide a foundation for future functional studies and may facilitate the development of molecular tools for improving N-use efficiency and yield performance in soybean improvement programs. Nevertheless, the candidate genes identified in this study should be considered putative, as they were selected based on genomic proximity to significant SNPs and prioritized according to their functional annotations and reported roles in stress signaling, N metabolism, oxidative stress responses, and plant development. Future studies integrating local linkage disequilibrium analyses, transcriptomic data, and functional validation will be necessary to identify the causal genes underlying the detected associations.

## 5. Conclusions

This study investigated the genetic basis of chlorophyll accumulation and seed weight in a soybean diversity panel evaluated under N− and N+ conditions. Significant phenotypic variation and moderate-to-high heritability were observed for both traits, supporting the use of BLUP-derived phenotypes for GWAS. The analysis identified 20 significant SNP markers associated with SPAD AUC N−, SW N−, and SW N+. No significant associations were detected for SPAD AUC under N+ conditions. Several candidate genes were associated with signal transduction, transcriptional regulation, and oxidative stress responses, suggesting their possible roles in N-responsive growth and development. Allelic effect analyses also showed significant phenotypic differences between alternative alleles at representative loci. Collectively, these findings improve our knowledge of the genetic mechanisms underlying soybean responses to N availability and provide candidate loci and genes that may facilitate the development of soybean cultivars with improved N-use efficiency and yield performance.

## Figures and Tables

**Table 1:** Descriptive statistics, including mean, standard deviation (SD), minimum (Min), maximum (Max), and coefficient of variation (CV), for SPAD area under the curve (SPAD AUC) and 1000-seed weight (SW) evaluated under nitrogen-deficient (N−) and nitrogen-sufficient (N+) conditions across 193 soybean accessions.

Trait	Mean	SD	Min	Max	CV%
SPAD AUC N−	728.78	129.80	144.80	994.25	17.81
SPAD AUC N+	782.88	97.92	564.75	1065.15	12.51
SW N−	116.51	33.78	35.9	278.5	28.99
SW N+	118.94	35.56	42.6	287.20	29.89

**Table 2:** Significant SNPs for SPAD area under the curve (SPAD AUC) and 1000-seed weight (SW) under nitrogen-deficient (N−) and nitrogen-sufficient (N+) conditions, chromosome (Chr.), physical position based on the soybean reference genome, *p*-value, and −log10(*p*).

Trait	SNP	Chr.	Position (bp)	*p*-Value	−log10(*p*)
SPAD AUC N−	ss715624313	16	30,674,962	1.12 × 10^−12^	9.95
SPAD AUC N−	ss715617226	13	13,787,354	1.13 × 10^−10^	9.95
SPAD AUC N−	ss715609999	11	28,353,697	1.57 × 10^−10^	9.80
SPAD AUC N−	ss715596121	7	144,508	3.81 × 10^−10^	9.42
SPAD AUC N−	ss715625979	17	12,875,585	9.20 × 10^−8^	7.04
SPAD AUC N−	ss715624813	16	36,146,766	1.50 × 10^−7^	6.83
SW N−	ss715582932	2	42,915,643	2.54 × 10^−11^	10.60
SW N−	ss715631103	18	46,130,859	7.18 × 10^−8^	7.14
SW N−	ss715612654	12	36,308,899	2.45 × 10^−7^	6.61
SW N−	ss715603222	9	1,904,113	4.23 × 10^−7^	6.37
SW N+	ss715587094	4	13,213,821	1.21 × 10^−13^	12.92
SW N+	ss715588530	4	47,858,859	4.54 × 10^−10^	9.34
SW N+	ss715596677	7	17,786,785	1.60 × 10^−8^	7.79
SW N+	ss715578470	1	1,301,020	1.72 × 10^−8^	7.77
SW N+	ss715623347	16	106,132	6.81 × 10^−8^	7.17
SW N+	ss715627827	17	41,223,428	7.81 × 10^−8^	7.11
SW N+	ss715591352	5	36,820,301	1.08 × 10^−8^	6.97
SW N+	ss715606637	10	38,553,575	1.16 × 10^−8^	6.94
SW N+	ss715623458	16	1,431,551	6.39 × 10^−8^	6.19
SW N+	ss715614171	13	25,259,816	7.39 × 10^−8^	6.13

**Table 3:** Candidate genes located within ±100 kb of significant SNPs for SPAD area under the curve (SPAD AUC) and 1000-seed weight (SW) under nitrogen-deficient (N−) and nitrogen-sufficient (N+) conditions, gene annotations, biological relevance, and prioritization.

Trait	SNP	Chr.	Candidate Gene	Annotation	Biological Relevance	Priority
SPAD AUC N-	ss715624313	16	*Glyma.16G145700*	Transmembrane protein 115-like/rhomboid-domain protein	Membrane-associated protein processing and stress-related signaling; possible role in chloroplast or membrane stress adaptation	Moderate
SPAD AUC N−	ss715617226	13	*Glyma.13G044600*	Alpha/beta superfamily hydrolase	Broad metabolic regulation; possible involvement in lipid or hormone-related stress-response pathways	Moderate
SPAD AUC N−	ss715609999	11	*Glyma.11G202600*	2-oxoglutarate (2OG) and Fe(II)-dependent oxygenase superfamily protein	Oxidoreductase activity and 2OG-dependent metabolism; biologically relevant to nitrogen/carbon metabolism and stress adaptation	Moderate-High
SPAD AUC N−	ss715596121	7	*Glyma.07G147900*	LRR receptor-like kinase family protein	Environmental signal perception and phosphorylation-mediated stress/nutrient signaling	Very High
SPAD AUC N−	ss715625979	17	*Glyma.17G153400*	Protein kinase superfamily protein	Signal transduction and phosphorylation-mediated regulation of growth and nutrient/stress responses	High
SPAD AUC N−	ss715624813	16	*Glyma.16G200300*	Peroxidase superfamily protein	Reactive oxygen species homeostasis and oxidative-stress tolerance; relevant to chlorophyll accumulation under low nitrogen	High
SW N−	ss715582932	2	*Glyma.02G240100*	Acyl-CoA synthetase 5	Fatty acid metabolism, lipid biosynthesis, and carbon allocation during seed development	Moderate-High
SW N−	ss715631103	18	*Glyma.18G191100*	MYB/SANT-like DNA-binding domain protein	Transcriptional regulation of development and stress-responsive gene networks	High
SW N−	ss715612654	12	*Glyma.12G202700*	EPIDERMAL PATTERNING FACTOR 1-like protein	Cell-to-cell signaling and epidermal/stomatal development; possible links to water-use efficiency and growth	High
SW N−	ss715603222	9	*Glyma.09G023600*	Homeodomain/START-domain DNA-binding protein	Transcriptional and developmental regulation with lipid-binding START domain; relevant to organ development and stress response	High
SW N+	ss715587094	4	*Glyma.04G115500*	WRKY family transcription factor	Transcriptional regulation of stress, defense, and nutrient-response pathways; strong candidate for nitrogen-related growth regulation	Very High
SW N+	ss715588530	4	*Glyma.04G206100*	Phosphatidylinositol-4-phosphate 5-kinase	Phosphoinositide signaling, membrane trafficking, and stress-response signaling	High
SW N+	ss715596677	7	*Glyma.07G148000*	Phosphoglucan water dikinase, chloroplastic-like isoform	Starch metabolism and carbohydrate partitioning; relevant to seed filling and seed weight	High
SW N+	ss715578470	1	*Glyma.01G013500*	Receptor kinase 3	Signal perception and phosphorylation-mediated developmental/environmental signaling	High
SW N+	ss715623347	16	*Glyma.16G001500*	Protein kinase superfamily protein	Protein phosphorylation and stress/growth signaling	High
SW N+	ss715627827	17	*Glyma.17G257700*	C2H2-like zinc finger protein	Transcriptional regulation and metal-ion binding; possible role in stress-responsive gene expression and development	High
SW N+	ss715591352	5	*Glyma.05G179900*	Ethylene-responsive element binding factor 1	Ethylene signaling and transcriptional regulation; likely involvement in abiotic-stress and nutrient-response pathways	Very High
SW N+	ss715606637	10	*Glyma.10G150200*	Phospholipase D P2	Lipid signaling, membrane remodeling, and abiotic-stress response	High
SW N+	ss715623458	16	*Glyma.16G015700*	ORMDL family protein	Membrane and lipid homeostasis; potential role in cellular signaling and stress adaptation	Moderate
SW N+	ss715614171	13	*Glyma.13G139800*	ATP-binding protein serine/threonine kinase	Signal transduction, phosphorylation cascades, growth regulation, nutrient sensing, and stress response	Very High

## Data Availability

The raw data supporting the conclusions of this article are available from the corresponding author upon request.

## Supplementary Materials

The following supporting information can be downloaded at: https://www.mdpi.com/article/10.3390/genes17080924/s1, Table S1. Soybean accessions included in the genome-wide association study, with cultivar name, country of origin, and maturity group.

## References

[1] Hartman G.L., West E.D., Herman T.K. (2011). Crops that Feed the World 2. Soybean-Worldwide Production, Use, and Constraints Caused by Pathogens and Pests. Food Secur..

[2] Ciampitti I.A., Salvagiotti F. (2018). New Insights into Soybean Biological Nitrogen Fixation. Agron. J..

[3] Cafaro La Menza N., Monzon J.P., Lindquist J.L., Arkebauer T.J., Knops J.M.H., Unkovich M., Specht J.E., Grassini P. (2020). Insufficient Nitrogen Supply from Symbiotic Fixation Reduces Seasonal Crop Growth and Nitrogen Mobilization to Seed in Highly Productive Soybean Crops. Plant Cell Environ..

[4] de Borja Reis A.F., Moro Rosso L., Purcell L.C., Naeve S., Casteel S.N., Kovács P., Archontoulis S., Davidson D., Ciampitti I.A. (2021). Environmental Factors Associated with Nitrogen Fixation Prediction in Soybean. Front. Plant Sci..

[5] Kubar M.S., Shar A.H., Kubar K.A., Rind N.A., Ullah H., Kalhoro S.A., Wang C., Feng M., Gujar A., Sun H. (2021). Optimizing Nitrogen Supply Promotes Biomass, Physiological Characteristics and Yield Components of Soybean (*Glycine max* L. Merr.). Saudi J. Biol. Sci..

[6] Wang W., Chen D., Sun H., Kant S., Hammond J.P., Shi L., Chu C. (2026). Genetic Improvement of Nitrogen- and Phosphorus-Use Efficiency in Crops: Old Goals with New Aspirations. Mol. Plant.

[7] Li S., Huang Y.Z., Liu X.Y., Fu X.D. (2021). Genetic Improvement of Nitrogen Use Efficiency in Crops. Yi Chuan.

[8] Tam V., Patel N., Turcotte M., Bossé Y., Paré G., Meyre D. (2019). Benefits and Limitations of Genome-Wide Association Studies. Nat. Rev. Genet..

[9] Hwang E.Y., Song Q., Jia G., Specht J.E., Hyten D.L., Costa J., Cregan P.B. (2014). A Genome-Wide Association Study of Seed Protein and Oil Content in Soybean. BMC Genom..

[10] Patel J., Patel S., Cook L., Fallen B.D., Koebernick J. (2025). Soybean Genome-Wide Association Study of Seed Weight, Protein, and Oil Content in the Southeastern USA. Mol. Genet. Genom..

[11] Yang C., Yan J., Jiang S., Li X., Min H., Wang X., Hao D. (2022). Resequencing 250 Soybean Accessions: New Insights into Genes Associated with Agronomic Traits and Genetic Networks. Genom. Proteom. Bioinform..

[12] Steketee C.J., Schapaugh W.T., Carter T.E., Li Z. (2020). Genome-Wide Association Analyses Reveal Genomic Regions Controlling Canopy Wilting in Soybean. G3.

[13] Dhanapal A.P., Ray J.D., Singh S.K., Hoyos-Villegas V., Smith J.R., Purcell L.C., Fritschi F.B. (2016). Genome-Wide Association Mapping of Soybean Chlorophyll Traits Based on Canopy Spectral Reflectance and Leaf Extracts. BMC Plant Biol..

[14] Liyanage D.K., Torkamaneh D., Belzile F., Balasubramanian P., Hill B., Thilakarathna M.S. (2023). The Genotypic Variability among Short-Season Soybean Cultivars for Nitrogen Fixation under Drought Stress. Plants.

[15] Li Y.Y., Ming B., Fan P.P., Liu Y., Wang K.R., Hou P., Li S.K., Xie R.Z. (2022). Effects of Nitrogen Application Rates on the Spatio-Temporal Variation of Leaf SPAD Readings on the Maize Canopy. J. Agric. Sci..

[16] Kim T.-H., Kim S.-M. (2024). Effects of SPAD Value Variations according to Nitrogen Application Levels on Rice Yield and Its Components. Front. Plant Sci..

[17] Xiong D., Chen J., Yu T., Gao W., Ling X., Li Y., Peng S., Huang J. (2015). SPAD-Based Leaf Nitrogen Estimation Is Impacted by Environmental Factors and Crop Leaf Characteristics. Sci. Rep..

[18] Galić V., Mazur M., Brkić A., Brkić J., Jambrović A., Zdunić Z., Šimić D. (2020). Seed Weight as a Covariate in Association and Prediction Studies for Biomass Traits in Maize Seedlings. Plants.

[19] Song Q., Hyten D.L., Jia G., Quigley C.V., Fickus E.W., Nelson R.L., Cregan P.B. (2013). Development and Evaluation of SoySNP50K, a High-Density Genotyping Array for Soybean. PLoS ONE.

[20] Purcell S., Neale B., Todd-Brown K., Thomas L., Ferreira M.A., Bender D., Maller J., Sklar P., de Bakker P.I., Daly M.J. (2007). PLINK: A Tool Set for Whole-Genome Association and Population-Based Linkage Analyses. Am. J. Hum. Genet..

[21] Alexander D.H., Novembre J., Lange K. (2009). Fast Model-Based Estimation of Ancestry in Unrelated Individuals. Genome Res..

[22] Wang J., Zhang Z. (2021). GAPIT Version 3: Boosting Power and Accuracy for Genomic Association and Prediction. Genom. Proteom. Bioinform..

[23] Malek M.A., Rafii M.Y., Afroz M.S.S., Nath U.K., Mondal M.M. (2014). Morphological Characterization and Assessment of Genetic Variability, Character Association, and Divergence in Soybean Mutants. Sci. World J..

[24] Sulistyo A., Purwantoro, Mejaya M.J., Nugrahaeni N., Suhartina (2021). Determination of Genetic Parameters of Seed Characteristics in Edible Soybean. Legume Res..

[25] Lee S., Van K., Sung M., Nelson R., LaMantia J., McHale L.K., Mian M.A.R. (2019). Genome-Wide Association Study of Seed Protein, Oil and Amino Acid Contents in Soybean from Maturity Groups I to IV. Theor. Appl. Genet..

[26] Qiang B., Chen S., Fan Z., Cao L., Li X., Fu C., Zhang Y., Jin X. (2025). Effects of Nitrogen Application Levels on Soybean Photosynthetic Performance and Yield: Insights from Canopy Nitrogen Allocation Studies. Field Crops Res..

[27] Salvagiotti F., Cassman K.G., Specht J.E., Walters D.T., Weiss A., Dobermann A. (2008). Nitrogen Uptake, Fixation and Response to Fertilizer N in Soybeans: A Review. Field Crops Res..

[28] Vogel J.T., Liu W., Olhoft P., Crafts-Brandner S.J., Pennycooke J.C., Christiansen N. (2021). Soybean Yield Formation Physiology—A Foundation for Precision Breeding Based Improvement. Front. Plant Sci..

[29] Sakuraba Y. (2022). Molecular Basis of Nitrogen Starvation-Induced Leaf Senescence. Front. Plant Sci..

[30] Krapp A. (2015). Plant Nitrogen Assimilation and Its Regulation: A Complex Puzzle with Missing Pieces. Curr. Opin. Plant Biol..

[31] Hao Q.N., Zhou X.A., Sha A.H., Wang C., Zhou R., Chen S.L. (2011). Identification of Genes Associated with Nitrogen-Use Efficiency by Genome-Wide Transcriptional Analysis of Two Soybean Genotypes. BMC Genom..

[32] Napier J.D., Heckman R.W., Juenger T.E. (2023). Gene-by-Environment Interactions in Plants: Molecular Mechanisms, Environmental Drivers, and Adaptive Plasticity. Plant Cell.

[33] Hirel B., Bertin P., Quilleré I., Bourdoncle W., Attagnant C., Dellay C., Gouy A., Cadiou S., Retailliau C., Falque M. (2001). Towards a Better Understanding of the Genetic and Physiological Basis for Nitrogen Use Efficiency in Maize. Plant Physiol..

[34] Soltabayeva A., Dauletova N., Serik S., Sandybek M., Omondi J.O., Kurmanbayeva A., Srivastava S. (2022). Receptor-like Kinases (LRR-RLKs) in Response of Plants to Biotic and Abiotic Stresses. Plants.

[35] Romero-Hernandez G., Martinez M. (2022). Plant Kinases in the Perception and Signaling Networks Associated with Arthropod Herbivory. Front. Plant Sci..

[36] Liu S., Wu Y., Qiu Y., He D., Zhao K., Yang L., Li Y., Zhang Z., Zou H., Liu Y. (2025). Genome-Wide Analysis of Class III Peroxidase Gene Family in *Glycine max* and Functional Roles in Stress Response. Sci. Rep..

[37] Borella J., Becker R., Lima M.C., de Oliveira D.d.S.C., Braga E.J.B., de Oliveira A.C.B., do Amarante L. (2019). Nitrogen Source Influences the Antioxidative System of Soybean Plants under Hypoxia and Re-Oxygenation. Sci. Agric..

[38] Yu Y., Wang N., Hu R., Xiang F. (2016). Genome-Wide Identification of Soybean WRKY Transcription Factors in Response to Salt Stress. SpringerPlus.

